# Public health round-up

**DOI:** 10.2471/BLT.19.010119

**Published:** 2019-01-01

**Ebola response continues despite attacks**

As the Ebola outbreak in the Democratic Republic of the Congo approaches seven months since being declared, the World Health Organization (WHO), working in collaboration with the Ministry of Health and partners, remains focused on containment and response in Butembo, Katwa, Beni and Kalunguta and other parts of the country. This photograph shows a WHO infection, prevention and control team at work in Beni, disinfecting houses and health facilities where people have died of Ebola.

**Figure:**
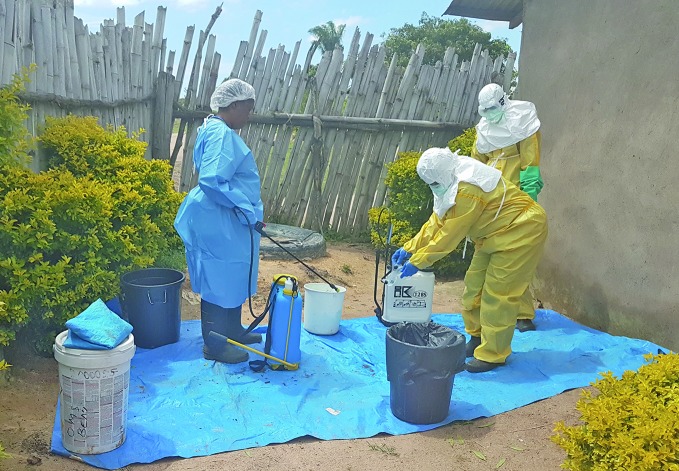
WHO/Eugene Kabambi

## First randomized control trial for Ebola treatments

The first-ever randomized control trial to evaluate the effectiveness and safety of four drugs used in the treatment of Ebola patients began 26 November 2018.

The multi-drug trial is being undertaken in the Democratic Republic of the Congo and is part of a multi-outbreak, multi-country study that was agreed by partners under a World Health Organization (WHO) initiative.

The trial started with three of the four treatments (Zmapp, mAb114, and remdesivir) at the Ebola treatment centre in the north eastern town of Beni. Future trials may include the REGN-EB3 antibody.

Prior to the launch of the trial, patients were treated under a compassionate use protocol, with therapeutics that have shown promise and which have a good safety profile in laboratory conditions. Some 160 patients have been treated in this way under an ethical framework called the Monitored Emergency Use of Unregistered and Investigational Interventions. The data in the North Kivu outbreak will likely be insufficient to draw firm conclusions about the treatments. It will therefore be necessary to extend the trials over a period of several years and multiple outbreaks.

The current trial is coordinated by WHO and led and sponsored by the Democratic Republic of the Congo’s National Institute for Biomedical Research, in partnership with its Ministry of Health, the United States’ National Institutes of Health, the Alliance for International Medical Action and other organizations.

http://www.who.int/news-room/detail/26-11-2018-democratic-republic-of-the-congo-begins-first-ever-multi-drug-ebola-trial

## Royalty-free licence for key hepatitis C treatment

A new royalty-free licence agreement has been signed between the Medicines Patent Pool (MPP) and pharmaceutical company AbbVie for glecaprevir/pibrentasvir (G/P). News of the agreement was announced by the MPP on 12 November.

G/P is an oral combination medicine that needs to be taken only once a day and has achieved cure rates as high as 98% in non-cirrhotic patients across all genotypes of the virus. WHO recommends G/P as a first-line treatment for people with hepatitis C infections, including those with renal impairment.

To date, access to the medicine has been limited because of its high cost. The licence will enable manufacturers to develop and sell affordable generic versions of the medicine in 99 low- and middle-income countries.

Globally, some 71 million people are currently living with chronic hepatitis C infections, of which an estimated 20% (14.2 million) have been diagnosed, while only 7% (5 million) are currently receiving treatment.

https://medicinespatentpool.org/mpp-media-post/the-medicines-patent-pool-signs-licence-with-abbvie-to-expand-access-to-key-hepatitis-c-treatment-glecaprevirpibrentasvir/

## Measles cases rise

Reported measles cases spiked in 2017, as multiple countries experienced severe and protracted outbreaks of the disease.

According to a report published in WHO’s *Weekly Epidemiological Record* and in the Centers for Disease Control’s *Morbidity and Mortality Weekly Report* on 29 November, measles cases increased 31% globally between 2016 and 2017, rising from 132 328 reported cases in 2016 to 173 330. 

However, the biggest increases occurred in the Americas, the Eastern Mediterranean Region, and Europe.

“The increase in measles cases is deeply concerning, but not surprising,” said Dr Seth Berkley, CEO of Gavi, the Vaccine Alliance. 

“Complacency about the disease and the spread of falsehoods about the vaccine in Europe, a collapsing health system in Venezuela and pockets of fragility and low immunization coverage in Africa are combining to bring about a global resurgence of measles after years of progress.”

“Existing strategies need to change: more effort needs to go into increasing routine immunization coverage and strengthening health systems. Otherwise we will continue chasing one outbreak after another,” he added.

Measles is a serious and highly contagious disease that can cause complications, including encephalitis, severe diarrhoea and dehydration, pneumonia, ear infections and permanent vision loss. 

Babies and young children with malnutrition and weak immune systems are particularly vulnerable to complications and death. 

The disease is preventable through two doses of a safe and effective vaccine.

http://www.who.int/news-room/detail/29-11-2018-measles-cases-spike-globally-due-to-gaps-in-vaccination-coverage

**Cover photo**

Iraq Yazidi boys, who have re-entered Iraq from the Syrian Arab Republic after escaping conflict in Sinjar District, find water in the town of Faysh Kabour, Dohuk Governorate.

**Figure:**
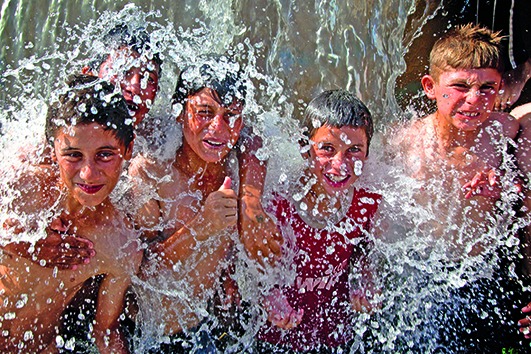
UNICEF/UNI169165/Wathiq Khuzaie

## Access issues hamper polio eradication

A panel of experts convened by WHO recommended that WHO continue to designate the international spread of poliovirus a Public Health Emergency of International Concern.

The committee expressed concern regarding the increase in wild poliovirus cases globally in 2018, especially in Afghanistan, where the number of polio cases almost doubled in 2018, with 19 cases reported as of 30 November, compared to 10 at the same time in 2017.

The Emergency Committee under the International Health Regulations (2005) (IHR) regarding the international spread of poliovirus cited the security situation, as well as persistent refusal to be vaccinated in some communities as obstacles to immunization campaigns.

Professor Helen Rees, chair of the committee, urged that all countries and partners regard polio eradication as an emergency. 

“We have the tools, we need to focus on what works, we need to get to every child,” she said. “The reality is that there is no reason why we should not be able to finish this job, but we have to keep at it.”

http://www.who.int/news-room/detail/30-11-2018-statement-of-the-nineteenth-ihr-emergency-committee-regarding-the-international-spread-of-poliovirus

## Country-led initiative to tackle malaria

WHO and partners have launched a country-led initiative to scale up prevention and treatment interventions to combat malaria in response to a reversal of declining cases.

According to the latest *World malaria report 2018*, an estimated 219 million people contracted malaria in 2017, compared to 217 million in 2016 and 214 million in 2015. 

The number of people contracting malaria globally had been steadily falling, from 239 million in 2010. 

If current trends continue, targets set by the *WHO Global technical strategy for malaria 2016–2030* to reduce malaria case incidence and death rates by at least 40% by 2020 will not be met.

The country-driven plan, *High burden to high impact: a targeted malaria response*, follows a call from WHO Director-General, Tedros Adhanom Ghebreyesus, for an aggressive new approach to galvanise the global malaria response. 

Launched on 19 November, the plan is designed to support countries with a high burden of malaria.

In 2017, approximately 70% of all malaria cases (151 million) and deaths (274 000) were concentrated in 11 countries, 10 of which are in Africa (Burkina Faso, Cameroon, Democratic Republic of the Congo, Ghana, Mali, Mozambique, Niger, Nigeria, Uganda and United Republic of Tanzania). 

An estimated 3.5 million more malaria cases were reported in these 10 African countries in 2017 compared to the previous year. 

http://www.who.int/news-room/detail/19-11-2018-who-and-partners-launch-new-country-led-response-to-put-stalled-malaria-control-efforts-back-on-track

## Change in treatment for drug-resistant tuberculosis

Injectable agents are no longer among the priority medicines for longer multidrug-resistant tuberculosis (MDR–TB) regimens and fully oral regimens should become the preferred option for most patients, according to updated WHO treatment guidelines. 

The new recommendations, published on 15 December, signal a departure from previous approaches to treating the disease.

About 600 000 new cases of MDR–TB are estimated to emerge each year, requiring treatment with second-line TB treatment regimens. 

Improvements in diagnostics and treatment have led to increased detection and cure rates, but many MDR–TB patients do not receive the treatment they need.

Three medicines – fluoroquinolones (levofloxacin or moxifloxacin), bedaquiline and linezolid – are strongly recommended for use in a longer regimen, which is completed with other medicines. 

Most regimens would include at least four agents, likely to be effective in the first 6 months and three thereafter.

The standardized, shorter MDR-TB regimen may be offered to eligible patients who agree to a briefer treatment (9-12 months) that may be less effective than an individualized longer regimen and that requires a daily injectable agent for at least four months. 

http://www.who.int/features/qa/79/en/

**Looking ahead**

28 January-5 February – 144th session of the Executive Board WHO headquarters.

29 January-3 February – The Prince Mahidol Award Conference, Bangkok, Thailand. Theme: The Political Economy of NCDs: A Whole of Society Approach.

11 February – Meeting on global mental health advocacy and communications WHO headquarters.

12–13 February – The First Food and Agricultural Organization/WHO/African Union International Conference on Food Safety.

